# The Increased Release Kinetics of Quercetin from Superparamagnetic Nanocarriers in Dialysis

**DOI:** 10.3390/antiox12030732

**Published:** 2023-03-16

**Authors:** Lucija Mandić, Marija Matković, Goran Baranović, Suzana Šegota

**Affiliations:** 1 Ruđer Bošković Institute, Division of Physical Chemistry, 10000 Zagreb, Croatia; 2 Ruđer Bošković Institute, Division of Organic Chemistry and Biochemistry, 10000 Zagreb, Croatia

**Keywords:** release kinetics, superparamagnetism, stationary magnetic field, alternating magnetic fields, magnetic nanoparticles, quercetin, viscosity

## Abstract

The actual cumulative mass of released quercetin from nanoparticles within the dialysis membrane was determined under the influence of external stationary and alternating magnetic fields. We have shown that the control of the release kinetics of quercetin from MNPs, i.e., the distribution of quercetin between the nanoparticles and the suspension within the membrane, can be tuned by the simple combination of stationary and alternating magnetic fields. Under non-sink conditions, the proportion of quercetin in the suspension inside the membrane is increased toward the nanoparticles, resulting in the increased release of quercetin. The results obtained could be applied to the release of insoluble flavonoids in aqueous suspensions in general.

## 1. Introduction

Nanoparticles can be designed as carriers that can bind or incorporate specific biomolecules with therapeutic properties, thus avoiding conventional drug resistance mechanisms [1,2,3,4]. Magnetic nanoparticles that show exceptional potential in biomedicine are superparamagnetic iron nanoparticles (SPIONs) (magnetite and maghemite) [5,6,7,8,9,10,11]. The localization of drugs with superparamagnetic magnetite nanocarriers in combination with an external magnetic field and their retention until the end of therapy represent a promising strategy for controlled drug delivery. Due to the presence of van der Waals and magnetic dipole–dipole attractive forces, magnetite nanoparticles interact with each other and start to form aggregates [12,13,14,15]. This problem can be avoided by functionalizing the surface of the nanoparticles [16,17]. To reduce the tendency to aggregate, protect their surface from oxidation, and make the whole system stable and biocompatible, magnetite nanoparticles are coated [18,19]. Poly(ethylene glycol) (PEG) is one of the most commonly used coating materials [5,10,20,21,22,23].

Quercetin, a dietary flavonoid and polyphenol, is found in plants, vegetables, and fruits [24,25]. In recent decades, quercetin has been considered one of the leading antioxidants among flavonoids and one of the most widely studied flavonoids [24]. However, the use of quercetin as a drug molecule in pharmaceutical studies is limited due to its instability, hydrophobic nature, poor solubility, and low bioavailability [22,23]. Encapsulation into suitable therapeutic nanosystems can lead to improved solubility, reduced toxic side effects, and enhanced pharmacokinetic and pharmacodynamic properties in addition to protecting the drug from degradation [24,25].

In numerous experiments measuring the release kinetics of drugs from nanocarriers, dialysis membranes are used to separate NPs from the released free drug. Although several recent papers on nanoparticle drug release acknowledge this problem, many other researchers have ignored the implications of using a dialysis bag to evaluate nanoparticle drug release kinetics. Thus, the measured drug mass outside the membrane does not correspond to the mass of the drug released from the nanoparticles inside the membrane. Additional diffusion barriers affecting the kinetics of drug release from nanoparticles were solved by Washington mathematical formulas in which the steady-state hypothesis assumes that the rate of drug release from nanoparticles and the rate of dialysis through the membrane are equivalent [26]. In this model, the drug release rate is determined by the volumes inside and outside the membrane, the membrane transfer rate, and the partition coefficient, i.e., the distribution of the drug between the nanoparticles and its continuous phase. Two cases are considered: (i) transport across the membrane follows first-order kinetics, and (ii) drug release is driven by diffusion from nanoparticles.

The purpose of this study was to investigate the transport of quercetin across the membrane and establish whether or not transport across the membrane is the rate-limiting factor and whether or not the release of quercetin is driven by diffusion from nanoparticles. To this end, experiments were performed using two different membranes (8 kD and 25 kD) and two media with different viscosities (PBS/EtOH (Vol.% 50:50) and 2-propanol/H_2_O (Vol.% 65:35)).

The actual cumulative mass of released quercetin from the nanoparticles within the dialysis membrane was determined under the influence of external stationary and alternating magnetic fields. We demonstrated that the control of the release kinetics of quercetin from the MNPs, i.e., the distribution of quercetin between the nanoparticles and solution inside the membrane, can be realized by a simple combination of stationary and alternating magnetic fields. With the application of external magnetic fields, the distribution of quercetin between the solution inside the membrane and nanoparticles increased, and, as expected, the increased release of quercetin was observed. The obtained results can be used in research on the controlled release of flavonoids in general.

In this study, we synthesized biocompatible MNPs with colloidal stability, high loading capacity, superparamagnetic properties, and negligible toxicity, which enable the delivery of unstable and water-insoluble quercetin. Moreover, MNPs can achieve maximum efficiency and the prolonged release of quercetin by low stationary and high-frequency alternating magnetic fields, showing their great potential as a promising drug delivery system for clinical therapy. Complementary therapies with iron chelators are an effective means to reduce the toxicity and aggregation of MNPs in clinical use and to eliminate iron overload [27].

Recently, it has been reported that, in addition to quercetin, many flavonoids with iron-chelating and antioxidant activities can reduce iron deposition and inhibit the lipid peroxidation caused by iron overload [28]. The simultaneous treatment of flavonoids as iron chelators with magnetite NPs not only reduces the toxicity of NPs but also increases the bioavailability of flavonoids. In addition, many flavonoids exhibit prooxidant activity upon prolonged treatment [28]. The balance between oxidant and antioxidant activities, i.e., the optimal mass of flavonoids [29,30], depends on the mass of the flavonoid and the duration of flavonoid exposure. Therefore, knowledge of the actual mass of the released drug reduces the overdose of flavonoids, eliminating their prooxidant effect, and this is crucial for the clinical application of a nano-delivery system.

## 2. Materials and Methods

### 2.1. Chemicals

Iron (III) chloride hexahydrate (97%) was purchased from Alfa Easar (Ottawa, ON, Canada). Ammonium acetate and poly(ethylene glycol) (PEG, Mw = 4000 Da) were purchased from Sigma Aldrich (St. Louis, MO, USA). Ethylene glycol and ethanol (96%) were purchased from Lach-ner (Neratovice, Czech Republic). Compressed nitrogen was purchased from Messer (Bad Soden am Taunus, Germany). Silicon oil was received from Acros organics (Waltham, MA, USA). Quercetin (≥99%) was supplied by Lach-ner (Neratovice, Czech Republic). 2-Propanol was purchased from Lach-ner (Neratovice, Czech Republic). Dialysis bags with molecular weight cut-offs (MWCOs) of 8 kDa and 25 kDa were purchased from Thermo Fisher Scientific (Waltham, MA, USA).

### 2.2. Synthesis of Superparamagnetic Magnetite Nanoparticles

Superparamagnetic magnetite nanoparticles were prepared by a modified solvothermal method using iron (III) chloride hexahydrate (97%), ammonium acetate, poly(ethylene glycol) (PEG, M_w_ = 4000 Da), and ethylene glycol. The detailed procedure for the synthesis of magnetite nanoparticles is described in our previous work [23].

### 2.3. Instrumentation and Material Characterization

X-ray powder diffraction with monochromatic CuKα radiation (*λ* = 0.154 nm) at room temperature using a Philips MPD 1880 diffractometer (Brooklyn, NY, USA) was used to analyze structural properties. A field-emission scanning electron microscope, JEOL JSM -7000F (Tokyo, Japan), was used to determine the morphology and surface texture of the magnetite MNPs. FTIR spectra were acquired with an Alpha-T FTIR spectrometer (Bruker, Billerica, MA, USA). At a nominal resolution of 4 cm^−1^ and a temperature of 25 °C, all spectra were recorded between 4000 and 350 cm^−1^, and the total number of recordings was 16. The dried samples were mixed with KBr powder and then pressed into pellets.

### 2.4. Loading of Quercetin into MNPs

A UV/VIS spectrophotometer was used to study the quercetin loading efficiency of the synthesized MNPs. The absorbance of the supernatant was measured at 374 nm using a WTW photoLab^®^ 7600 UV/VIS spectrophotometer (Xylem, Rye Brook, NY, USA) to determine the loading efficiency. Measurements were performed in a rectangular quartz cuvette with covers and an optical path length of 10 mm at a temperature of 25 °C. In an ultrasonic bath (Bandelin Sonorex Super RK 100 H, Berlin, Germany), 500 mg of quercetin was dissolved in 100 mL of ethanol and suspended in the bath for one hour at room temperature. Then, 100 mg of coated MNPs and a 25 mL aliquot of the quercetin solution were added to a 50 mL Falcon centrifuge conical tube. The mixture was mechanically stirred for 24 h at 30 rpm and 25 °C in a digital rotator (IKA LOOPSTER digital, IKA Works GmbH & Co, Staufen, Germany). Then, the quercetin-loaded MNPs and the unloaded quercetin were separated by centrifugation (Universal 320 Hettich Zentrifugen, Tuttlingen, Germany) at 7500 rpm for 10 min. The loading efficiency was calculated according to the following equation:

(1) $$LE=\frac{m_{embedded}}{m_{NP}}\times 100\%$$

where *m_embedded_* is the mass of quercetin incorporated into the nanoparticles, and *m_NP_* is the total mass of bare MNPs used for loading.

### 2.5. Release Study of Quercetin under Stationary and Alternating Magnetic Fields

The setup of the apparatus is the same as in our previous study [23]. Briefly, the combination of stationary (11.0 mT) and alternating magnetic fields (50 kHz and 100 kHz), perpendicular to each other, at controlled electric current (I = 100 mA) allows the control of the kinetic profile of quercetin release. The high-frequency alternating magnetic field was chosen, in agreement with Brezovitch, without any deleterious effects on the organism [27]. A total of 100 mg of the quercetin-loaded MNPs was transferred to a dialysis bag (MWCOs 8 kDa and 25 kDa, Thermo Fisher Scientific (Waltham, MA, USA)) and added to volumes of 1 mL and 5.5 mL, respectively. The dialysis bag was placed in a thermostated reactor at a physiological temperature (37 °C) with a stirrer set at 200 rpm. At specific time intervals, 1 mL of the supernatant was replaced with a fresh medium. The media used were PBS/EtOH (Vol.% 50:50) and 2-propanol/H_2_O (Vol.% 65:35).

At frequencies of alternating magnetic fields (50 and 100 kHz), the release of quercetin would be significantly higher than that without magnetic fields.

The drug release was calculated by measuring the absorbance of quercetin released in the supernatant at *λ* = 330 nm for the PBS/EtOH medium (Vol.% 50:50) and at *λ* = 374 nm for the 2-propanol/H_2_O medium (Vol.% 65:35) using a UV/VIS spectrophotometer. The linearity of the calibration covers the concentration range from 1 × 10^−6^ to 1 × 10^−4^ mol dm^−3^ (*r*~1.0). All release kinetics experiments were performed at least in triplicate, and the results are presented as averages with calculated standard deviations.

### 2.6. Dynamic and Electrophoretic Light Scattering

The hydrodynamic diameter (*d*_H_) of the suspended nanoparticles and the zeta potential (*ζ*) were measured using the M3-PALS technique with a photon correlation spectrophotometer (Zetasizer Nano ZS, Malvern, UK), with a green laser (λ = 532 nm). All measurements were performed at 25 °C in 2-propanol/H_2_O (Vol.% 65/35). The hydrodynamic diameter was determined using the peak maximum of the volume size distribution. Using a Smoluchowski approximation (f(κa) = 1.5), the zeta potential (*ζ*) was calculated from the electrophoretic mobility. The average *d*_H_ values were calculated as the average of ten measurements and presented as the average of the MNP population, while the zeta potential values were calculated as the average of three independent measurements. Zetasizer 6.32 software was used to acquire the results (Malvern, UK).

### 2.7. Viscosity Measurements

Measurements were performed with a Lovis 2000 M rolling-ball viscometer (Anton Paar, Austria) using a long capillary with a steel ball. Before the measurements, the instrument was adjusted on the surface with MQ H_2_O. The Lovis standard method was used, defined by the following parameters: measurement mode TTS (25 °C, 30 °C, 37 °C), hand angle 38°, measurement cycles 2, coefficient of variation 0.1%, measurement distance Automatic sample. Densities were determined by measuring the mass of the exact volume with an analytical balance (up to 0.01 mg).

The validity of the measurement was checked by comparing the determined viscosity values of water and 2-propanol with the known values at 25 °C (0.89 mPa s and 2.07 mPa s, respectively) [31]. The values agreed very well.

### 2.8. Kinetics

**Figure 1:**
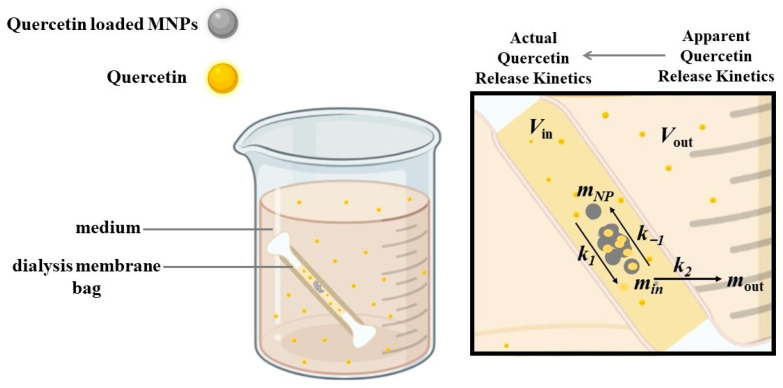
Experimental setup for studying the quercetin release kinetics under non-sink conditions.

The experimental setup is shown in Figure 1. The volume *V*_in_ (donor volume) inside the bag contains the drug carrier suspension in which equilibrium was established between the drug adsorbed on the nanoparticles and the drug released into the donor volume. Thus, the experiment was conducted under non-sink conditions. Washington established mathematical formulas in 1989 that predicted how an additional diffusion barrier would affect the kinetics of drug release from nanoparticles [26]. Although several recent papers on drug release from nanoparticles acknowledge this problem, many other researchers have disregarded the effects of using a dialysis bag when evaluating the kinetics of drug release from nanoparticles.

The volume *V*_out_ (receiver volume, *V*_out_ >> *V*_in_) on the other side of the dialysis bag contains a sink phase in which the drug concentration is always far from saturation, although not close to zero. For our experiment, where the masses of released quercetin are reported, it was more convenient to rewrite the formulas adopted by Yu et al. [32] in terms of masses *m* rather than concentrations *c*.

In our release kinetics experiments, we made the following assumptions:

(i) The total mass of quercetin remained nearly constant throughout the release experiment because the amount of drug repeatedly removed from the uptake volume for concentration measurements was always negligible;

(ii) Both the solution inside the bag and the solution outside the membrane were homogeneous due to constant stirring;

(iii) The volume inside the membrane *V*_in_ and the volume outside *V*_out_ were assumed to be constant (model II in Yu et al. [32]). In addition, *V*_in_ = 4.7 mL (mean value) and *V*_out_ = 150 mL, i.e., *V*_in_/*V*_out_ = 0.03 << 1.

The equations to start from are:

(2) $$\frac{dm_{in}}{dt}=r\left(t\right)-\frac{AP}{V_{in}}m_{in}+\frac{AP}{V_{out}}m_{out}$$

(3) $$\frac{dm_{out}}{dt}=\frac{AP}{V_{in}}m_{in}-\frac{AP}{V_{out}}m_{out}$$

where *r*(*t*) represents the source of drug molecules (quercetin-loaded MNPs) and measures the rate of the mass release of the drug from MNPs. It has the dimension kg s^−1^. We are interested in the (fractional) quantity of the mass of the quercetin released into the membrane interior (*m*_in_) and measured in the membrane exterior (*m*_out_) at time *t*, which is given by

(4) $$f\equiv \frac{1}{m_{out,0}}\int_{0}^{t}r\left(t\right)dt=\frac{m_{in}}{m_{out,0}}+\frac{m_{out}}{m_{out,0}}$$

where *m*_out,0_ is the total mass of quercetin released. It is different from the total mass of quercetin on nanocarriers imported in the bag (see below). However, *f*, which is also known as the drug release fraction from the formulation in the membrane interior, can hardly be directly measured. What is measured is the apparent rate of release, defined by

(5) $$f_{app}=\frac{m_{out}\left(t\right)}{m_{out,0}}$$

and *f* can be expressed in terms of *f*_app_:

(6) $$f=\left(1+\frac{V_{in}}{V_{out}}\right)f_{app}+\frac{1}{k_{cal}}\frac{df_{app}}{dt}$$

where *k*_cal_ is obtained from calibration measurements, whereby the function

(7) $$m_{cal}\left(t\right)=m_{cal,0}\left(1-e^{-k_{cal}t}\right)$$

is fitted to the results of calibration measurements (quercetin is in the dialysis bag, and there are no nanocarriers), and the quantities *m*_cal,0_ and *k*_cal_ are obtained.

The apparent release rate *f*_app_ = *m*_out_/*m*_0_ was parametrized by fitting the Weibull function:

(8) $$m_{out}\left(t\right)=m_{out,0}\left(1-e^{-at^{b}}\right)$$

(*m*_out,0_ in Equation (8) is not *m*_cal,0_ in Equation (6)). In Equation (7), the positive parameters *a* and *b* are not given any physical interpretation, although the values of *b* can be related to the drug release mechanism [33]. The data analysis was conducted by using the nonlinear least-squares optimization routine from R-package stats [34].

After measuring *f*_app_, we are in a position to explore the function *f* of the actual release rate from the MNPs themselves given in Equation (5). It is useful to discuss the behavior of *f* in two limiting cases, i.e., when *t*→∞ and when *t*→0. It is easily seen that *f*→1 + *V*_in_/*V*_out_ for *t*→∞, and thus, it is always greater than one:

(9) $$\frac{m_{in,t\to \infty }}{m_{out,0}}=\frac{V_{in}{\left[C_{in}\right]}_{t\to \infty }}{V_{out}{\left[C_{out}\right]}_{0}}=\frac{V_{in}}{V_{out}}$$

In other words, the final concentrations in the bag and in the receiver volume are the same:

(10) $$\frac{{\left[C_{in}\right]}_{t\to \infty }}{{\left[C_{out}\right]}_{0}}=1$$

as it should be since the concentration difference is the driving force of the diffusion across the membrane. However, if *m*_total_ is the total mass of quercetin in the system and *m*_NP_ is the mass of unreleased quercetin, then

*m*_total_ = *m*_NP_(*t*) + *m*_in_(*t*) + *m*_out_(*t*) (11)

At *t* = 0, *m*_total_ = *m*_NP_(0) + *m*_in_(0) + *m*_out_(0) = *m*_NP_(0) because *m*_in_(0) = 0 and *m*_out_(0) = 0. When *t*→∞,

(12) $$m_{total}=m_{NP,t\to \infty }+m_{in,t\to \infty }+m_{out,0}=m_{NP,t\to \infty }+\left(1+\frac{V_{in}}{V_{out}}\right)m_{out,0}$$

The model thus predicts the incomplete release of quercetin. This would mean that the equilibrium between loaded NPs and quercetin dissolved in *V*_in_ is characterized by the final concentration [C_in_]_,*t*→∞_ = *m*_in,*t*→∞_/*V*_in_. Following Washington, 1989, a partition coefficient, *K*_p_, is introduced:

(13) $$K_{p}=\frac{m_{in}}{m_{NP}}$$

It is a time-independent constant if the diffusion across the membrane is slow so that *m*_NP_ and *m*_in_ are in equilibrium from the very beginning of the measurement. Thus,

(14) $$m_{total}=\frac{1}{K_{p}}m_{in,t\to \infty }+\left(1+\frac{V_{in}}{V_{out}}\right)m_{out,0}$$

so that

(15) $$\frac{m_{out,0}}{m_{total}}=\frac{1}{1+\left(1+\frac{1}{K_{p}}\right)\frac{V_{in}}{V_{out}}}$$

i.e.,

(16) $$\frac{1}{K_{p}}=-1+\frac{V_{out}}{V_{in}}\left(\frac{m_{total}}{m_{out,0}}-1\right)$$

The limiting values for *t*→0 depend on *b*:

(i) For *b* = 1, the shape of the curve corresponds exactly to the shape of an exponential profile with the constant *k* = 1/*a*;

(ii) For *b* > 1, *f*→0, but it has a maximum at some time *t*: i.e., it increases up to that time and then decreases;

(iii) For *b* < 1, *f*→∞, and it decreases in the whole interval (0, +∞).

### 2.9. Calculation of Standard Deviations

All data values are reported as the average ± SD. The standard deviation based on a sample is calculated using the formula

(17) $$SD=\sqrt{\frac{\sum_{i}{\left(x_{i}-\overset{=}{x}\right)}^{2}}{\left(n-1\right)}}$$

where *x*_i_ is the sample value, $\overset{=}{x}$ is the sample average, and *n* is the sample size.

## 3. Results and Discussion

### 3.1. Characterization of Synthesized Magnetite MNPs

Magnetite nanoparticles (MNPs) functionalized with the polymer polyethylene glycol (PEG) were prepared by the solvothermal method, as reported in a previous study [23]. Briefly, a mixture of 1.35 g FeCl_3_ × 6H_2_O, 3.85 g CH_3_COONH_4_, 1.0 g PEG (M = 4 kDa), and 70 mL ethylene glycol was vigorously stirred at 160 °C for 1 h under an inert nitrogen atmosphere. After cooling to room temperature, the mixture was heated to 200 °C in an autoclave for 19 h and then cooled down to 50 °C. The MNPs were separated from the solvent by centrifugation at 8000 rpm for 10 min, washed several times with ethanol, and dried for further characterization.

**Figure 2:**
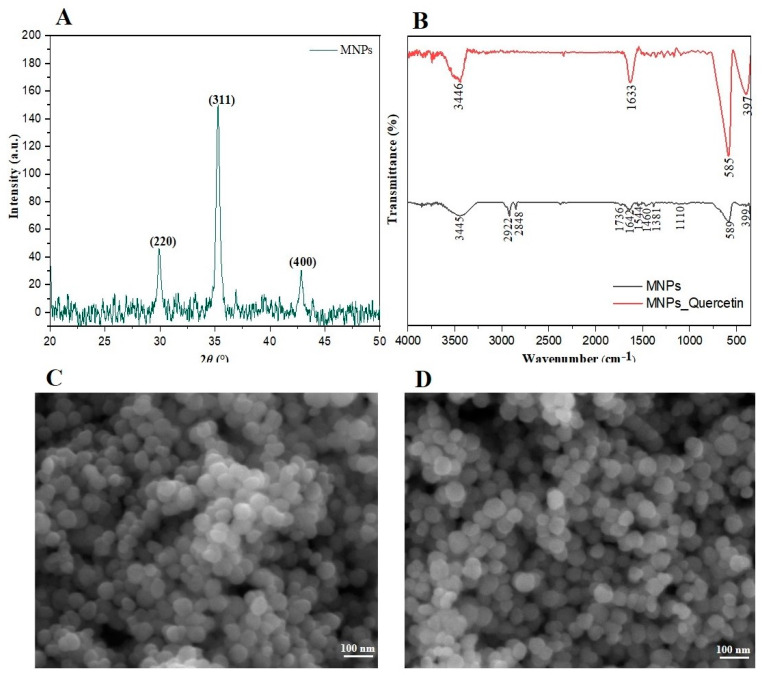
(**A**) XRPD analysis of bare MNPs; (**B**) FTIR spectrum of bare and quercetin-loaded MNPs; (**C**) SEM images of bare MNPs and (**D**) quercetin-loaded MNPs.

Figure 2A shows the X-ray powder diffraction patterns of the synthesized magnetite MNPs. The XRPD patterns’ distinctive peaks match the magnetite diffraction peaks, confirming the cubic inverse spinel structure of MNPs. The sharp diffraction of three characteristic peaks, (220), (311), and (400), also indicate the spinel structure of magnetite. The FTIR spectrum (Figure 2B) of MNPs shows bands at 589 cm^−1^ and 399 cm^−1^, corresponding to the symmetric stretching vibration mode of the Fe-O bond. The XRPD patterns’ distinctive peaks match the magnetite diffraction peaks, confirming the cubic inverse spinel structure of MNPs, which exhibits the widening of the OH band at 3446 cm^−1^. The loading efficiency was calculated from 15 independent experiments and amounted to (19.2 ± 1.9)%.

FE-SEM was performed to analyze the shape and morphology of the prepared magnetite MNPs (Figure 2C) and quercetin-loaded MNPs (Figure 2D). The SEM images confirm a uniform spherical shape, with partial agglomeration due to magneto–dipole interactions between MNPs. The electron microscopy images confirmed that bare and quercetin-loaded MNP nanoparticles were uniform spheres with similar dimensions.

Li et al. reported a simple solvothermal approach to synthesize monodisperse superparamagnetic iron oxide NPs in ethylene glycol. The results show that the prepared monodisperse spherical Fe_3_O_4_ NPs have a diameter range of 80–100 nm [35]. Recently, Wang et al. reported a modified solvothermal method in which the average sizes of iron oxide nanoparticles were under the control of the mass ratio of Na_3_Cit/NaOAc, and the results showed that Fe_3_O_4_ nanoparticles had a spherical shape and an almost uniform particle diameter, whose value was 135 nm [36]. Based on Li’s and Wang’s findings, the solvothermal method produces uniform, spherical iron oxide NPs with diameters ranging from 80 to 140 nm, which is consistent with our results.

### 3.2. Viscosity Measurements

The dynamic viscosity data for water and the solvent mixtures PBS/EtOH (Vol.% 50:50) and 2-propanol/H_2_O (Vol.% 65:35) (Table 1) at temperatures of 25 °C, 30 °C, and 37 °C are shown in Table 1.

The measurements of dynamic viscosity at lower temperatures (namely, 25 °C and 30 °C, since no accurate or relevant data are available for 37 °C) were performed to verify the validity of the measurements. A comparison of the determined viscosity values of water and 2-propanol with the known values at 25 °C (0.89 mPa s and 2.07 mPa s, respectively) [31] was performed to ensure that the measurements were as accurate as possible and in order to check repeatability and accuracy.

For all the measured samples, the dynamic viscosity decreases with increasing temperature. The results for water are in agreement with the literature data (*η*(H_2_O, 25 °C) = 0.891 mPa s (mean value from [37,38,39]), indicating the accuracy and validity of our measured values. The value measured for the 2-propanol/H_2_O mixture (*η*(2-propanol/H_2_O, *x*(2-propanol) = 0.304, 25 °C) was 3.08 mPa s, which agrees with the literature value of 3.068 mPa s determined by Pang et al. (2007) [40].

### 3.3. Dynamic and Electrophoretic Light Scattering Experiment

The stability and homogeneous dispersion of MNPs were investigated by dynamic and electrophoretic light scattering measurements and are presented in Table 2.

The zeta potential of MNPs in aqueous suspensions above *ζ* = ±30 mV appears to be relevant to the stability and pharmacokinetics of NPs [41]. In our previous work, we determined the zeta potential of MNPs in phosphate-buffered solution (PBS/EtOH, Vol.% 50:50) to be *ζ* = (−30.6 ± 0.7) mV, *ζ* = (−35.1 ± 1.5) mV, and *ζ* = (−31.3 ± 0.8) mV for bare, PEG-coated, and quercetin-loaded MNPs, respectively [23]. In this work, the zeta potential was used to investigate the stability of colloidal suspensions of PEG-coated MNPs and quercetin-loaded MNPs in a medium with increased dynamic viscosity, namely, 2-propanol/H_2_O (vol.% 65:35). Therefore, zeta potential values differed from those measured in the PBS/EtOH (Vol.% 50:50) buffer solution. Bare MNPs showed a zeta potential *ζ* = (−6.1 ± 0.3) mV, whereas quercetin-loaded MNPs slightly decreased the absolute zeta potential value to *ζ* = (−3.1 ± 0.3) mV. However, the change in zeta potential did not significantly affect the stability of the MNPs in the 2-propanol/H_2_O mixture (Vol.% 65:35).

Dynamic light scattering measurements showed that the average hydrodynamic diameter of the quercetin-loaded MNP population was smaller (*d*_Have_ = (953 ± 366) nm) than that of the bare MNPs (*d*_H_ = (1602 ± 103) nm) with a narrower size distribution, indicating better colloidal stability with simultaneous quercetin loading and reduced aggregation in the 2-propanol/H_2_O (Vol.% 65:35) mixture. The unimodal volume size distributions of both samples and the polydispersity index of the bare MNPs (PDI = 0.38 ± 0.05) and quercetin-loaded MNPs (PDI = 0.2 ± 0.1) indicate that quercetin reduces the aggregation process and breaks the MNP cluster, which increases the stability of the MNPs in the 2-propanol/H_2_O (Vol.% 65:35) mixture.

### 3.4. Kinetics

#### 3.4.1. Influence of Membrane Molecular Weight on Dialysis Rate of Quercetin in PBS/EtOH

In accordance with the Washington model [26], the membrane is considered to be a crucial factor in drug dialysis, and here, we first attempted to answer the question of whether quercetin transfer across membranes with different pore sizes is the first-order rate-limiting factor in order to perform experiments on the release kinetics of MNP nanocarriers in the next step. In this context, we performed calibration experiments; i.e., we investigated the effect of the dialysis membrane molecular weight cut-off (MWCO) on drug diffusion across the membrane.

Since the MWCO of the dialysis membrane should be about 100 times larger than the size of the drug molecule [32], we used dialysis membranes with MWCOs of 8 and 25 kDa and compared the obtained values of their calibration constants. The results are shown in Table 3.

The calculated calibration constants in the PBS/EtOH medium (Vol.% 50:50) were *k*_cal_ = (1.1 ± 0.3) × 10^−3^ min^−1^ and *k*_cal_ = (9.2 ± 0.8) × 10^−3^ min^−1^ for 8 kDa and 25 kDa membranes, respectively. The determined release half-life values are *τ* = 909 min and *τ* = 108 min for 8 kDa and 25 kDa membranes, respectively. Comparing the constants in the calibration experiments, the diffusion constant of the 25 kDa membrane is higher but in the same order of magnitude as that of the 8 kDa membrane. The obtained results regarding the effect of the membrane on the quercetin release profile are in agreement with previously published studies using dialysis membranes with different MWCO values [26,42,43,44]. Using similar dialysis membranes with 2, 7, and 10 kDa MWCOs, Moreno-Bautista et al. obtained slower diffusion rates than those obtained with the higher MWCO used in this study [45].

**Figure 3:**
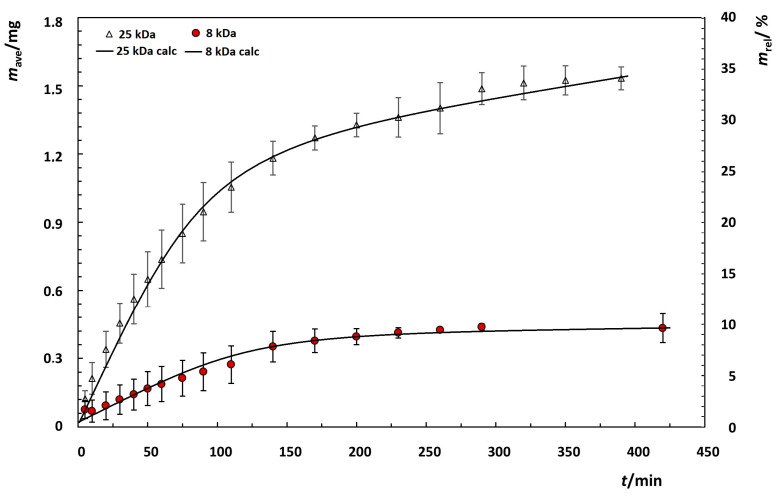
The cumulative mass of quercetin through dialysis membranes (8 kDa and 25 kDa) in PBS/EtOH (Vol.% 50:50) at a temperature of 37 °C (observed and calculated, Equation (7)) (left ordinate) and the fraction of dialyzed quercetin relative to the total mass of loaded quercetin contained in the dialysis bag at *t* = 0 (right ordinate).

The dialyzed mass of quercetin at a temperature of 37 °C over time is shown in Figure 3 (left ordinate), whereas the percentage of dialyzed quercetin relative to the total mass of quercetin introduced into the dialysis bag at *t* = 0 is shown in Figure 3 (right ordinate). At 400 min, the percentage of dialyzed quercetin across the 8 kDa membrane is only 10%, while for the 25 kDa membrane, it increases to 35%. As expected, the results show a higher rate constant across the 25 kDa membrane than across the 8 kDa membrane, and the results are consistent with Schwarzl’s findings [46].

#### 3.4.2. Influence of Media on the Dialysis

According to the Washington model [26], the rate of dialysis across the membrane is determined by the properties of the membrane itself, such as pore size, and this is a factor that has been the subject of numerous kinetic studies with nanocarriers that used membrane bags. However, for the application of nanocarriers, it is necessary to study drug release in media other than the commonly used buffers. Therefore, we used 2-propanol/H_2_O (Vol.% 65:35) as a model medium with a higher dynamic viscosity than PBS/EtOH (Vol.% 50:50).

Drug dialysis exhibits different kinetic shapes and rates depending on the viscosity of the medium [41]. The results of the determination of the calibration constants for the 25 kDa membrane at 37 °C are shown in Table 3. In the 2-propanol/H_2_O medium (Vol.% 65:35) for the 25 kDa membrane, *k*_cal_ = (5.47 ± 0.08) × 10^−3^ min^−1^ (release half-life is *τ* = 183 min), and as expected, the dialysis rate is lower than in the PBS/EtOH medium (Vol.% 50:50), which is also consistent with the viscosity measurements.

**Figure 4:**
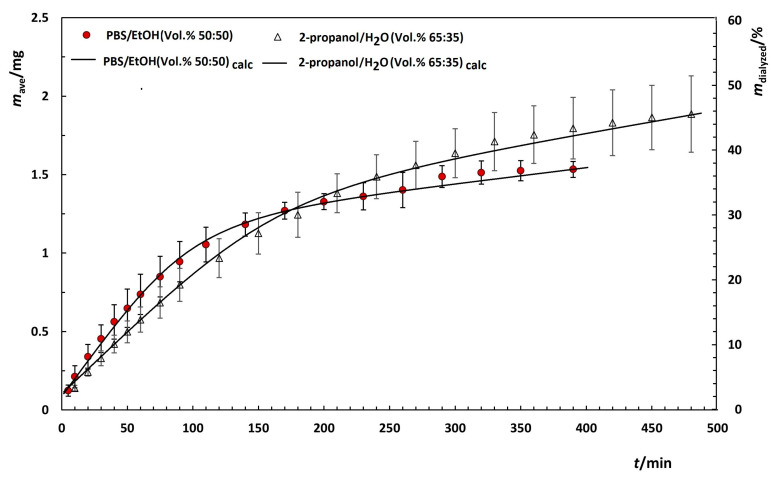
The cumulative mass of the quercetin through dialysis membranes with MWCO of 25 kDa in PBS/EtOH (Vol.% 50:50) and 2-propanol/H_2_O (Vol.% 65:35) at 37 °C with single exponential decay fit (left ordinate) and fraction of dialyzed quercetin relative to the total mass of quercetin in dialysis bag at *t* = 0 (right ordinate).

Let us now consider the kinetics of quercetin dialysis through the 25 kDa membrane shown in Figure 4. Although the experiments were performed with the same membrane and the same volume inside and outside the membrane, the kinetic profiles have different shapes, which are manifested in the different dialysis rates as a function of the viscosity of the medium [47].

During the first 200 min, the fraction of dialyzed quercetin relative to the total mass of quercetin is higher in the PBS/EtOH medium (Vol.% 50:50), although the solubility of quercetin is higher in 2-propanol/H_2_O (Vol.% 65:35) [48,49]. However, in the early stage of dialysis in 2-propanol/H_2_O (Vol.% 65:35), the mass fraction of dialyzed quercetin is lower than in the PBS/EtOH (Vol.% 50:50) medium because the retention time of quercetin within the membrane is higher due to the higher dynamic viscosity of the medium. This means that the quercetin molecules need more time to reach the membrane than in the PBS/EtOH (Vol.% 50:50) medium with a lower dynamic viscosity. On the other hand, the higher solubility of quercetin in 2-propanol/H_2_O (Vol.% 65:35) ensures dialysis under sink conditions, while dialysis in the PBS/EtOH (Vol.% 50:50) medium under non-sink conditions additionally involves the possible equilibrium between dissolved and undissolved quercetin. At 400 min, the higher solubility of quercetin in 2-propanol/H_2_O (Vol.% 65:35) leads to an increased mass fraction of dialyzed quercetin (35%) compared to that in PBS/EtOH (Vol.% 50:50) (10%). The obtained results show that the diffusion of quercetin is a rate-determining factor in dialysis under sink conditions, while dialysis under non-sink conditions is determined by the solubility of quercetin, resulting in an increased fraction of dialyzed quercetin.

#### 3.4.3. Kinetics of Quercetin Release from MNPs

**Figure 5:**
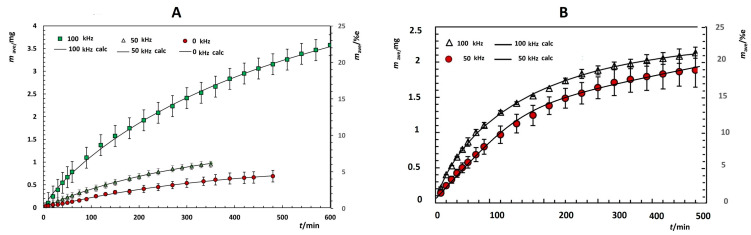
The cumulative mass of released quercetin through dialysis membranes with an MWCO of 25 kDa (left ordinate) at 37 °C and the fraction of dialyzed quercetin relative to the total mass of quercetin in nanocarriers at *t* = 0 (right ordinate) under a magnetic field *B* = 11 mT and alternating field with frequencies of 0 kHz, 50 kHz, and 100 kHz in (**A**) PBS/EtOH (Vol.% 50:50) and (**B**) 2-propanol/H_2_O (Vol.% 65:35).

Here, we attempted to determine the actual cumulative mass of quercetin inside the membrane using the model proposed by Yu et al. [32] for dialysis under non-sink conditions described by Washington [26]. The results are shown in Table 4 for the experiment performed at 37 °C without and with an applied external magnetic field in PBS/EtOH (Vol.% 50:50) and 2-propanol/H_2_O (Vol.% 65:35) media. In the case of the experiment performed without external magnetic fields in the PBS/EtOH (Vol.% 50:50) medium using an 8 kDa membrane, the actual mass of released quercetin in the membrane is almost equal to the maximum mass of quercetin at *t*→∞. The parameter *b* is slightly higher than 1, indicating that there is no burst effect and the release kinetics approaches the simple exponential release profile. This suggests that the rate constant of quercetin release from the nanoparticles is equivalent to the dialysis rate constant across the membrane. Our idea is based on the results of our previous study, in which we observed the increased release of quercetin when applying external magnetic fields. Therefore, we focused on how we can control the release kinetics of quercetin from the MNPs, i.e., how we can control the distribution of quercetin between the nanoparticles and the solution inside the membrane by applying a combination of stationary and alternating magnetic fields. When applying external magnetic fields, the distribution of quercetin between the suspension inside the membrane and the nanoparticles should be increased (due to the increased precession of the nanoparticles), and consequently, the equilibrium distribution of quercetin within the membrane suspension and within the nanoparticles should be shifted to the side of increased quercetin mass in the suspension, resulting in the increased release of quercetin. Indeed, the increased mass of released quercetin was observed when applying a combination of external magnetic fields in both PBS/EtOH (Vol.% 50:50) and 2-propanol/H_2_O (Vol.% 65:35) using an 8 kDa membrane. Compared with the released mass without a magnetic field (*m*_app_ = 0.70 ± 0.08) mg, the apparent quercetin mass increased to (*m*_app_ = 1.0 ± 0.2) and (*m*_app_ = 3.54 ± 0.3), respectively. The results are shown in Figure 5 as the average mass from at least three independent measurements.

**Figure 6:**
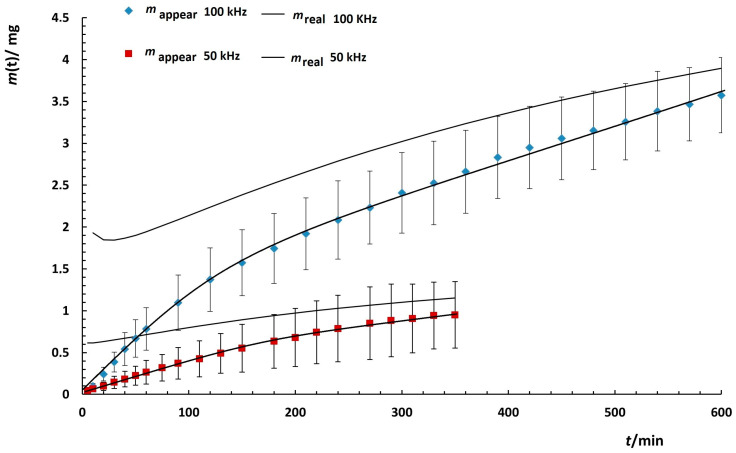
The cumulative apparent mass of released quercetin through 25 kDa dialysis membranes in PBS/EtOH (Vol.% 50:50) at 37 °C and calculated actual dialyzed mass of quercetin at 37 °C under a permanent magnetic field *B* = 11 mT and alternating field with frequencies of 50 and 100 kHz.

Similar results to those shown in Figure 5B were obtained in the 2-propanol/H_2_O (Vol.% 65:35) medium, but the effect of increasing the applied external magnetic field from a frequency of 50 kHz to 100 kHz is different in the different media. For example, the observed increase in released quercetin in the PBS/EtOH medium (Vol.% 50:50) is almost 100%, while in 2-propanol/H_2_O (Vol.% 65:35), only a 10% higher mass of quercetin is released when the frequency increases from 50 kHz to 100 kHz. This result suggests that the dialysis rate constant is determined by the diffusion of quercetin toward, but not across, the membrane. We were able to calculate the actual mass of quercetin released from the nanoparticles under these experimental conditions; the results are shown in Figure 6.

The difference between the apparent and actual masses of quercetin released is greater when the frequency of the applied alternating magnetic field is increased from 50 kHz to 100 kHz. Obviously, the difference is much higher at the beginning of the experiments and approaches the mass of released quercetin at *t*→∞ across the dialysis membrane with time. This result supports our prediction that at the beginning of the experiments, the rate constant of quercetin release from the nanoparticles is higher than the dialysis rate constant, leading to an accumulation of quercetin mass in the membrane. However, over time, a new equilibrium is established, leading to a decrease in the difference between the apparent and actual masses of quercetin released in the dialysis experiment.

The accumulation of quercetin within the membrane should also be mentioned here, even if it is assumed that the membrane is thin enough and the dialysis rate remains constant throughout the dialysis experiment. Moreover, in this type of dialysis experiment, there is a possibility that not only the quercetin but also the nanoparticles will accumulate. Therefore, the membrane pore size and other properties are key factors in performing high-quality dialysis experiments and should be selected very carefully.

In this work, it is shown that the release of quercetin from superparamagnetic nanoparticles is an efficient flavonoid delivery system, which is generally able to protect and deliver flavonoids and control their release by applying external weak magnetic fields [7,8,9]. Moreover, the application of superparamagnetic nanoparticles is characterized by the possibility of choosing the localization of drug therapy. In this way, the problem arising from the low solubility and instability of flavonoids as such drugs is largely circumvented. Moreover, superparamagnetic iron nanoparticles have been approved by the U.S. Food and Drug Administration (FDA). Therefore, their application extends not only to the field of biomedicine but also to the field of food to increase its nutritional value. The results obtained in this study could be applied to the release of insoluble flavonoids in aqueous suspensions in general.

## 4. Conclusions

Superparamagnetic magnetite nanoparticles (MNPs) were prepared by the solvothermal method and functionalized with the polymer poly(ethylene glycol) PEG-4000 Da. The XRPD patterns’ distinctive peaks confirmed the cubic inverse spinel structure of MNPs. The FTIR spectrum confirmed the loading of quercetin in MNPs.

The SEM images confirm a uniform spherical shape, with partial agglomeration due to magneto–dipole interactions between MNPs. Bare MNPs in the 2-propanol/H_2_O mixture (Vol.% 65:35) showed a zeta potential of *ζ* = (−6.1 ± 0.3) mV, whereas quercetin-loaded MNPs showed a slight decrease in the absolute zeta potential value to *ζ* = (−3.1 ± 0.3) mV. The unimodal volume size distributions of bare and quercetin-loaded MNPs and the polydispersity indexes of the bare MNPs (PDI = 0.38 ± 0.05) and quercetin-loaded MNPs (PDI = 0.2 ± 0.1) indicate the increased stability of the MNPs in the 2-propanol/H_2_O (Vol.% 65:35) mixture.

Calibration experiments were performed in order to investigate the effect of the dialysis membrane molecular weight cut-off (MWCO) on drug diffusion across the membrane. The obtained results show that the diffusion of quercetin is a rate-determining factor in dialysis under sink conditions, while dialysis under non-sink conditions is determined by the solubility of quercetin.

The calculated calibration constants in the PBS/EtOH medium (Vol.% 50:50) were *k*_cal_ = (1.1 ± 0.3) × 10^−3^ min^−1^ and *k*_cal_ = (9.2 ± 0.8) × 10^−3^ min^−1^ at 37 °C for 8 kDa and 25 kDa membranes, respectively. In the 2-propanol/H_2_O medium (Vol.% 65:35) with the 25 kDa membrane, *k*_cal_ = (5.47 ± 0.08) × 10^−3^ min^−1^ (release half-life is *τ* = 183 min), the dialysis rate is lower than in the PBS/EtOH medium (Vol.% 50:50) with higher dynamic viscosity.

The actual cumulative mass of released quercetin from the nanoparticles within the dialysis membrane was determined using the model for dialysis under non-sink conditions under the influence of external stationary and alternating magnetic fields. When applying external magnetic fields, the distribution of quercetin between the suspension inside the membrane and the nanoparticles is increased, and the equilibrium distribution of quercetin within the membrane suspension and within the nanoparticles is shifted to the side of increased quercetin mass in the suspension, resulting in the increased release of quercetin. With the application of external magnetic fields with a frequency increase from 50 kHz to 100 kHz, the observed increase in released quercetin in the PBS/EtOH medium (Vol.% 50:50) is almost 100%, and in 2-propanol/H_2_O (Vol.% 65:35), only a 10% higher mass of quercetin is released. The difference between the apparent and actual masses of released quercetin is greater at the beginning of the experiments when the frequency of the applied alternating magnetic field is increased from 50 kHz to 100 kHz. The rate constant of quercetin release from the nanoparticles is higher than the dialysis rate constant, leading to the accumulation of quercetin inside the membrane. Over time, a newly established equilibrium leads to a decrease in the difference between the apparent and actual masses of quercetin released in the dialysis experiment.

The results obtained in this study could be applied to the release of insoluble flavonoids in aqueous suspensions in general.

## Figures and Tables

**Table 1:** Dynamic viscosity of water and mixtures PBS/EtOH (Vol.% 50:50) and 2-propanol/H_2_O (Vol.% 65:35).

Sample	Dynamic Viscosity/mPa s		
	Temperature/°C		
	25	30	37
H_2_O	0.902 ± 0.007	0.812 ± 0.006	0.705 ± 0.006
PBS/EtOH (Vol.% 50:50)	2.38 ± 0.02	2.01 ± 0.02	1.61 ± 0.01
2-Propanol/H_2_O (Vol.% 65:35)	3.08 ± 0.03	2.57 ± 0.03	2.03 ± 0.02

**Table 2:** Average hydrodynamic diameter of population, polydispersity index (PDI), and zeta potential (*ξ*/mV) of bare and quercetin-loaded MNPs in a 2-propanol/H_2_O mixture (Vol.% 65:35) measured at 25 °C.

MNPs	*d*_Have_/nm	PDI	*ξ*/mV
bare MNPs	1602 ± 103	0.38 ± 0.05	−6.1 ± 0.3
Q-MNPs	953 ± 366	0.2 ± 0.10	−3.1 ± 0.3

**Table 3:** Rate constants in calibration experiments.

Medium	Membrane/kDa	10^3^ × *k*_cal_/min^−1^
PBS/EtOH (Vol.% 50:50)	8	1.1 ± 0.3
PBS/EtOH (Vol.% 50:50)	25	9.2 ± 0.8
2-Propanol/H_2_O (Vol.% 65:35)	25	5.47 ± 0.08

**Table 4:** Parameters of the Weibull function describing the release kinetics of quercetin through a 25 kDa membrane without and under a permanent magnetic field *B* = 11 mT and alternating magnetic field at frequencies of 0 kHz, 50 kHz, and 100 kHz at 37 °C in (A) PBS/EtOH (Vol.% 50:50) and (B) 2-propanol/H_2_O (Vol.% 65:35) media.

Medium	Frequency/ kHz	MWCO/ kDa	*a*/min^−1^	*b*	*^a^ m*_app_/mg	*^b^ m*_0_/mg
2-Propanol/H_2_O (Vol.% 65:35)	100	25	0.017 ± 0.001	0.80 ± 0.01	2.1 ± 0.1	2.34 ± 0.03
PBS/EtOH (Vol.% 50:50)	0	8	0.0021 ± 0.0003	1.06 ± 0.05	0.70 ± 0.08	0.91 ± 0.07
	50		0.0038 ± 0.002	1.10 ± 0.03	1.0 ± 0.2	1.5 ± 0.1
	100		0.0049 ± 0.0003	0.84 ± 0.02	3.54 ± 0.3	5.4 ± 0.4
	50	25	0.011 ± 0.002	0.93 ± 0.05	0.83 ± 0.03	0.87 ± 0.03
	100		0.010 ± 0.002	1.07 ± 0.04	0.706 ± 0.002	0.707 ± 0.006

*^a^ m*_app_—mass of released quercetin through dialysis membranes; *^b^* *m*_0_—maximum mass of released quercetin at *t*→∞ across dialysis membranes.

## Data Availability

The data presented in this study are available upon request from the corresponding author.
